# Prognosis of adenosquamous carcinoma of the pancreas

**DOI:** 10.1007/s00423-026-04035-0

**Published:** 2026-03-30

**Authors:** Shu Sasaki, Dimitri Chappalley, Lorenzo Bernardi, Fulvia Serra, Christine Sempoux, Antonia Digklia, Nermin Halkic, Emmanuel Melloul, Emilie Uldry, David Fuks, Gaëtan-Romain Joliat

**Affiliations:** 1 Department of Visceral Surgery, Lausanne University Hospital CHUV, Rue du Bugnon 46, Lausanne, CH-1011 Switzerland; 2 Faculty of Biology & Medicine (FBM), University of Lausanne (UNIL), Lausanne, Switzerland; 3 Department of Pathology, Lausanne University Hospital CHUV, Lausanne, Switzerland; 4 Department of Oncology, Lausanne University Hospital CHUV, Lausanne, Switzerland

**Keywords:** Pancreatic adenosquamous carcinoma, Pancreatic cancer, Pancreatectomy, survival.

## Abstract

**Purpose:**

Pancreatic adenosquamous carcinoma (PASC) is a rare malignant pancreatic tumor, defined as a pancreatic tumor containing at least 30% squamous cell carcinoma component. Most previous publications have reported a poor prognosis for patients with PASC, but data remain scant including clinicopathological features and treatment. This study aimed to characterize the clinicopathological features and to assess the survival of patients with PASC.

**Methods:**

A single center retrospective cohort study at the Lausanne University Hospital (CHUV) was performed. Patients pathologically diagnosed with PASC from January 1, 2010, to December 31, 2023, were included. Overall survival (OS) and disease-free survival (DFS) were calculated using the Kaplan-Meier methods and log-rank test was used for comparisons.

**Results:**

A total of 12 patients were histopathologically diagnosed with PASC. Median age at diagnosis was 67.5 years (IQR 59-75.5). Most patients were men (8/12). Eight patients underwent resection and 4 palliative chemotherapy or best supportive care. Six out of 8 patients underwent pancreatoduodenectomy. Median tumor size on pathological analysis was 4.8 cm (IQR 3.4–5.8). Median OS of the entire cohort was 9.2 months (95% CI 2.1–23.4). Median OS from time of diagnosis of patients with and without resection of PASC were 10.2 months (95% CI 2.4–24.1) vs. 8.8 months (95% CI 1.2–11.3, *p* = 0.23). Median DFS was 2.8 months (95% CI 1.5–23.4) for patients with resection.

**Conclusions:**

Patients with PASC had a very poor prognosis in this cohort. All patients who were not operated died before a year of the diagnosis.

## Introduction

Pancreatic adenosquamous carcinoma (PASC) is a rare malignant pancreatic tumor, accounting for 0.4% to 4% of all exocrine pancreatic tumors [1]. PASC clinically manifests similarly to pancreatic ductal adenocarcinoma (PDAC), including abdominal pain, jaundice, and weight loss [2]. Although there are some imaging features, suggestive of PASC on enhanced computed tomography and magnetic resonance imaging [3], the diagnosis of PASC relies on histopathological examination. According to the criteria of the World Health Organization, the proportion of squamous cell carcinoma component necessary for the diagnosis of PASC is at least 30% [4].

Most of the available published series reported a poor prognosis for patients with PASC with median overall survival (OS) ranging from 2 to 31 months depending on the type of performed treatments [5–10]. Studies that specifically analyzed the characteristics and clinical outcomes of PASC compared to PDAC showed controversial data regarding OS [5, 6, 11–13]. On the contrary, PASC tumors were often larger, more frequently poorly differentiated and with lymph node involvement, and more frequently involved the pancreatic tail compared to PDAC [5, 12].

Studies on PASC from registry or national database often lack data granularity and as the clinicopathologic features are not fully understood, case series with patient-level data can bring new information on this rare disease. The present cohort addresses this gap by providing detailed clinical information, including postoperative chemotherapy regimens, postoperative recurrence patterns, and detailed information on systemic treatments for non-resected patients, which have been noted in some prior studies but without detailed characterization. The aim of this study was to characterize the clinicopathological features and outcomes of patients with a histopathologically diagnosed PASC.

## Methods

The design of the study is a single-center retrospective cohort study. The inclusion criteria were age ≥ 18 years and patient pathologically diagnosed with PASC at the Lausanne University Hospital (CHUV) from January 1, 2010, to December 31, 2023. Patients without signature and acceptance of the CHUV general consent were excluded. Diagnosis was based on histopathological analysis (biopsy or surgical resection). The proportion of squamous cell carcinoma component necessary for the diagnosis of PASC was defined to be at least 30% [4]. The clinical characteristics collected were as follows: age, sex, comorbidities, clinical presentation, laboratory values, tumor markers, tumor location, tumor size, tumor histology, pathological features, oncological treatments, surgical data, and survival data. Cases were classified according to the TNM 8th edition staging manual of the American Joint Commission on Cancer [14]. R0 resection was defined based on a 1 mm tumor-free margin according to the British Royal College of Pathologists [15]. Statistical analyses were performed using JMP pro, version 17.0.0 (SAS Institute Inc., Cary, N.C.). The primary endpoint was median OS defined as the time from diagnosis or surgical resection of PASC to death. Secondary endpoints were median DFS and recurrence rates and locations of operated patients with PASC. Survival curves were performed using Kaplan-Meier technique and survivals were compared using the log-rank test. All patients were followed at our institution or affiliated centers, and no cases were lost to follow-up. The study was approved by the appropriate Research Ethics Committee (CER-VD, #2024 − 01362).

## Results

**Fig. 1:**
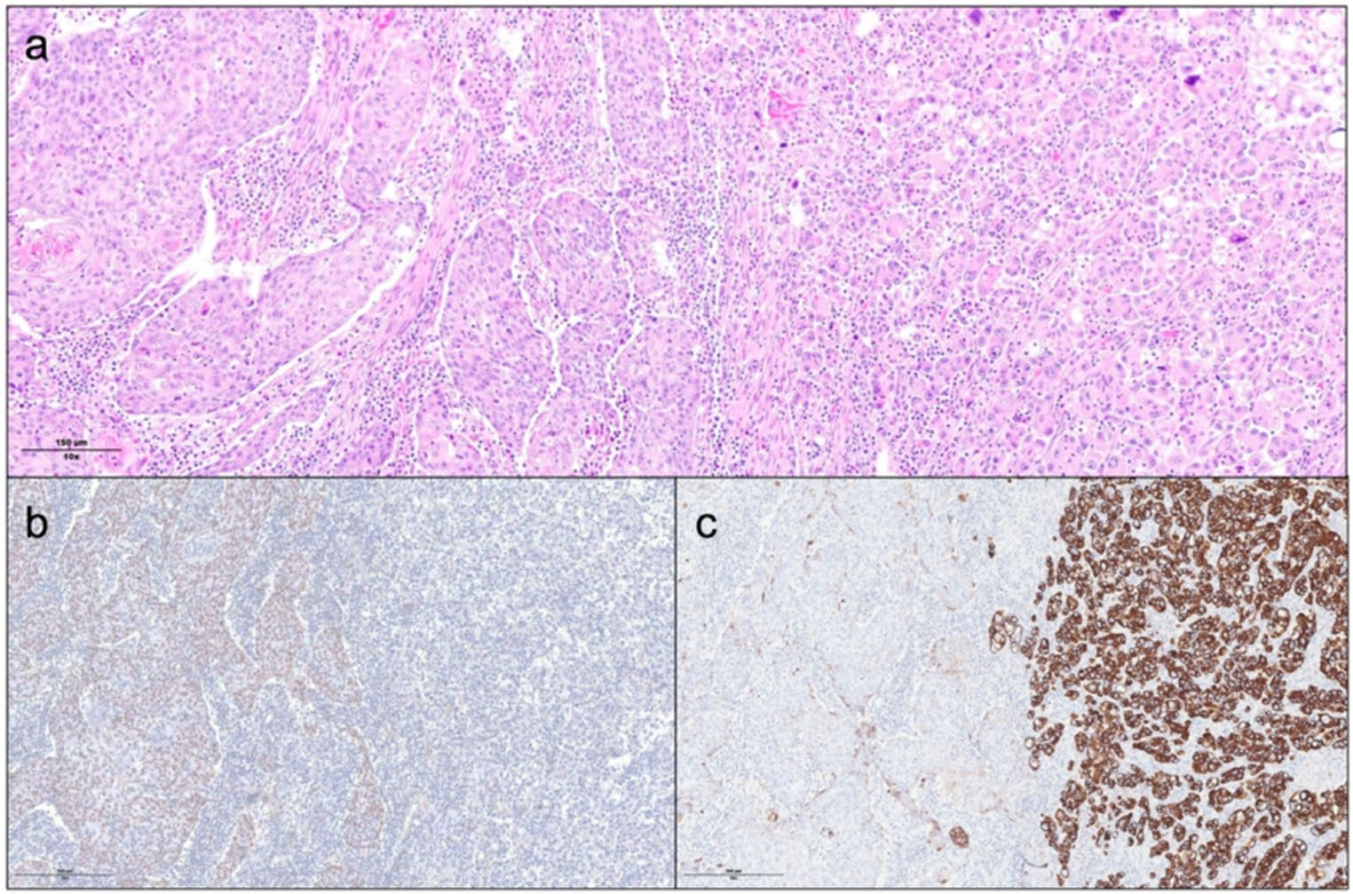
(**a**) hematoxylin-eosin staining, original magnification 10x, (**b)** p40 immunostaining underlying the squamous component on the left, original magnification 10x, (**c)** CK7 immunostaining underlying the poorly differentiated glandular component on the right, original magnification 10x

A total of 12 patients were histopathologically diagnosed with PASC between 2010 and 2023. Figure 1 shows typical histopathological image of PASC. Out of 521 resected pancreatic adenocarcinomas during the study period, 8 (1.5%) patients were confirmed to have PASC. Demographic and clinicopathological characteristics of patients with PASC (*n* = 12, 8 resected and 4 non operated) are summarized in Table 1. Median age at diagnosis was 67.5 years (IQR 59-75.5). Most patients were men (8/12). Six patients presented with abdominal pain and 2 patients presented with jaundice. Tumors were located in the head of pancreas in 8 patients and in the pancreas tail in 4 patients. Median tumor size on preoperative CT was 5.1 cm (IQR 3.1–5.5) and median initial CA 19 − 9 was 364 U/I (IQR 16-2730). Preoperative biliary drainage with plastic stent was performed in one patient for hyperbilirubinemia (total bilirubin level: 250 umol/l). At the time of diagnosis, 8 patients were judged as resectable (stage IB and IIA), 2 patients were locally advanced (stage III, tumor in contact with superior mesenteric artery > 180 degrees) and 2 patients had liver metastases (stage IV). The treatment strategy for PASC was upfront surgery in 8 patients. Two patients received FOLFIRINOX as a neoadjuvant chemotherapy but were not resected due to disease progression. One patient received gemcitabine and nab-paclitaxel as palliative chemotherapy. Best supportive care was provided for one patient with poor general condition. Preoperative details of patients who underwent pancreatectomy are summarized in Table 2.

**Table 1:** Demographic and clinical characteristics of patients diagnosed with pancreatic adenosquamous carcinoma (*n* = 12)

	Median with IQR or number with %
Age, years	67.5 (59-75.5)
Sex, n (%)	
Male	4 (33.3%)
Female	8 (66.7%)
Initial symptoms	
Abdominal pain	6 (50.0%)
Jaundice	2 (16.7%)
Weight loss	1 (8.3%)
Bleeding of the tumor	1 (8.3%)
Tumor anatomical location (head/tail)	8/4 (66.7%/33.3%)
Tumor size on CT, cm	5.1 (3.4–5.5)
Initial CA19-9, U/l	364 (23-3730)
Diabetes Mellitus	2 (16.7%)
Stage (AJCC 8^TH^ edition)	
IB	3 (25.0%)
IIA	5 (41.7%)
III	2 (16.7%)
IV	2 (16.7%)
Initial treatment	
Upfront resection	8 (66.7%
FOLFIRINOX as neoadjuvant chemotherapy	2 (16.7%)
GEM/nab-PAC as palliative chemotherapy	1 (8.3%)
BSC	1 (8.3%)
Preoperative biliary drainage	1 (8.3%)

*GEM/nab-PAC* gemcitabine and nab-paclitaxel, *BSC *best supportive care

**Table 2:** Overall demographic and clinical characteristics of the patients with resected pancreatic adenosquamous carcinoma (n=8)

	Median with IQR or number with %
Age, y, median	72 (61-79)
Sex, n (%)	
Male	2 (25.0%)
Female	6 (75.0%)
Tumor anatomical location (head/body/tail)	6/0/2 (75%/0%/25%)
Tumor size on CT, cm, median	4.6 (3.1-5.5)
Initial CA19-9, median	435 (16-2730)
Diabetes mellitus	1 (12.5%)
Surgical procedure (PD/DP)	6/2 (75%/25%)
Operation duration, minutes, median	374 (284-411)
Intraoperative blood loss, ml, median	800 (338-1250)
Artery reconstruction	0
Vascular reconstruction (PV/SMV/LRV)	3(1/1/1)
Pancreatic anastomosis (Pancreato-jejunal/ Pancreato-gastric)	6/0
Neoadjuvant therapy	0

*PD* Pancreatoduodenectomy, *DP* Distal Pancreatectomy, *PV* portal vein, *SMV*superior mesenteric vein, *LRV* left renal vein

**Fig. 2:**
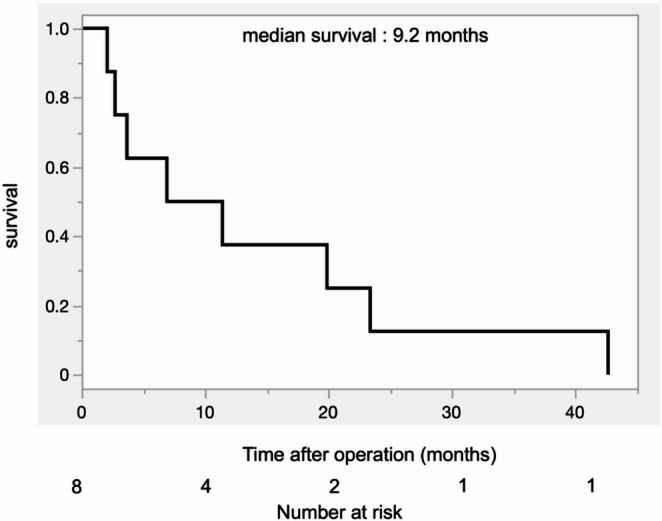
Overall survival of patients after resection of pancreatic adenosquamous carcinoma

**Fig. 3:**
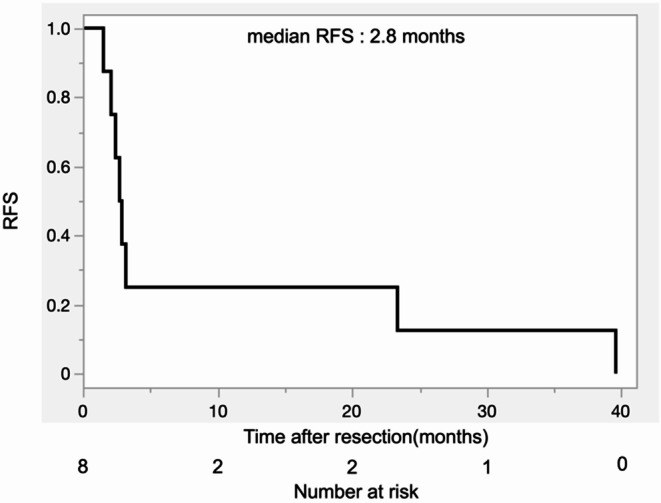
Recurrence-free survival of patients after resection of pancreatic adenosquamous carcinoma

Table 3 shows detailed clinical outcomes and histopathological features of each patient with resected PASC. Details of the adjuvant treatment regimen and on the recurrence and survival data of patients with resected PASC are summarized in Table 4. Six out of 8 patients underwent pancreatoduodenectomy. Distant metastases were found in 2 patients (liver and omentum) during operation and were resected concomitantly as the pancreatectomy. Two out of 8 patients experienced major postoperative complications (Clavien–Dindo grade ≥ III). Postoperative pancreatic fistula occurred in three patients, and four patients developed gastroparesis. No postoperative bleeding was observed. Lymph node metastases were positive in 6 patients. OS of patients with resected PASC is shown in Fig. 2 (median OS from time of surgery: 9.2 months, 95% CI 2.1–23.4). Of note, median OS of all patients with resected PDAC was 25 months (95% CI 20–30). The median OS for R0 patients was 9.2 months (95% CI 3.7–20.0) and the median OS for R1 patients was 13.0 months (95% CI 2.1–42.6) without significant difference (log-rank test *p* = 0.49). Seven patients had a recurrence and died of the disease, and one patient died of COVID-19 two months after the operation without recurrence. Liver metastases were observed in all 4 patients with known records of recurrence details. One patient had a local recurrence in the pancreas at the same time. The median DFS for the 8 patients was 2.8 months (95% CI 1.5–23.4, Fig. 3). 

**Table 3:** Specific surgical and pathological characteristics of each patient with pancreatic adenosquamous carcinoma who underwent resection

Case	Tumor location	Surgical procedure	Additional procedure	Operation Time (min)	Pathological staging *				L	Pn	V	LN Positive /Collected	*R*	G
					T	*N*	M	stage						
1	head	PD	No	417	3	1	0	IIB	1	1	1	2/28	0	3
2	head	PD	SMV resection	313	3	1	0	IIB	1	1	1	3/31	0	3
3	tail	DP	Left nephrectomy	228	3	1	0	IIB	0	1	1	2/14	0	3
4	head	PD	Wedge resection of hepatic metastases	431	3	1	1	IV	1	0	1	2/23	0	3
5	head	PD	PV resection	411	3	1	0	IIB	1	1	1	5/29	1	N/A
6	head	PD	No	374	2	0	0	IB	0	1	1	0/15	1	3
7	head	PD	No	284	2	1	0	IIB	1	1	1	4/23	1	3
8	tail	DP	Total gastrectomy, left adrenalectomy, segmental resection of the left colonic angle and wedge resection of the left renal vein.	380	3	0	1	IV	0	1	1	0/17	1	2

*PD* pancreatoduodenectomy, *DP* distal pancreatectomy, *SMV* superior mesenteric vein, *PV* portal vein, *L* lymph duct invasion, *Pn* perineural invasion, *V* vein invasion, *LN* lymph node, *R* resection margin, *G* tumor grade

* Classification of the 8th American joint committee on cancer (AJCC) staging for tumors of the pancreas

**Table 4:** Postoperative course of patients with resected pancreatic adenosquamous carcinoma

Case	Adjuvant therapy	Recurrence	Recurrence site	Number of recurrent tumors	Disease free survival, month	Survival until death, month	Cause of death
1	Gemcitabine	Yes	Liver	multiple	3	4	Carcinoma
2	mFOLFIRINOX	Yes	Liver	1	2	20	Carcinoma
3	mFOLFIRINOX	Yes	Liver, Pancreas	3	3	11	Carcinoma
4	FOLFIRI	Yes	Liver	8	2	7	Carcinoma
5	Gemcitabine	Yes	N/A	N/A	40	43	Carcinoma
6	No	Yes	N/A	N/A	N/A	24	Carcinoma
7	No	No	-	-	2	2	COVID-19
8	Unknown	Yes	N/A	N/A	N/A	3	Carcinoma

**Fig. 4:**
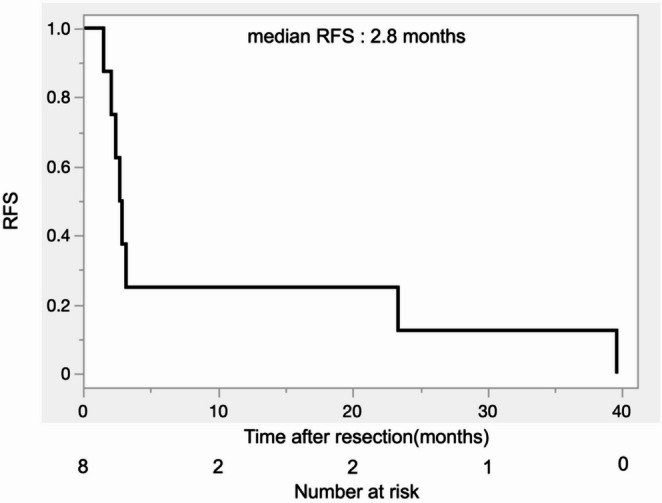
Overall survival of patients with and without resection of pancreatic adenosquamous carcinoma

 Table 5 shows detailed clinical outcome of patients without resection. Figure 4 shows OS from time of diagnosis of patients with resection and without resection of PASC (median OS: 10.2 months, 95% CI 2.4–24.1 vs. 8.8 months, 95% CI 1.2–11.3, log-rank test *p* = 0.23).

**Table 5:** Clinical outcomes of patients without resection of pancreatic adenosquamous carcinoma

Case	Tumor location	InitialCA199, U/l	Staging *				Treatment	Survival until death, month	Cause of death
			T	N	M	Stage			
9	head	89	4	0	0	III	1st FOLFIRINOX 6 cycles (3months) 2nd Gem/nab-PAC	7	Carcinoma
10	head	364	4	0	0	III	1st mFOLFIRINOX 1 cycle only due to intolerance 2nd Gem/Nab-PAC	11	Carcinoma
11	tail	38149	3	0	1	IV	BSC	1	Carcinoma
12	tail	23	4	0	1	IV	1st GEM/nab-PAC (5months) 2nd Cis/Gem/Nivolumab (4months)	11	Carcinoma

*GEM/nab-PAC* gemcitabine and nab-paclitaxel, *BSC *best supportive care, *Cis/Gem/Nivolumab* Cisplatin/Gemcitabine/ Nivolumab

* Classification of the 8th American joint committee on cancer (AJCC) staging for tumors of the pancreas

## Discussion

The incidence of PASC in our cohort was 1.5% of all resected pancreatic cancer patients. Moreover, median OS and DFS were 10.2 months (95% CI 2.4–24.1) and 2.8 months (95% CI 1.5–23.4) for operated patients. All patients who were not operated did not survive more than a year after diagnosis.

Clinical features such as age, sex ratio and initial symptoms in our cohort were similar to previous published cohorts [5, 8, 16, 17]. Boyd et al.. reported that compared to PDAC, PASC tumors were more frequently poorly differentiated, larger, and node positive [12]. In our cohort, 6 of 8 resected tumors were poorly differentiated, the median tumor size on preoperative CT was 4.6 cm, and 6/8 patients had lymph node involvement.

Regarding management, local control and aggressive resection have been recommended as the first-choice treatments for patients with PASC [18]. Surgical resection seems to be the best option for PASC patients who can achieve R0 resection [6, 11]. For advanced PASC, FOLFIRINOX and gemcitabine/nab-paclitaxel are both standard first-line treatments [19, 20]. In our cohort, 2 locally advanced patients received FOLFIRINOX and 1 with distant metastases received gemcitabine/nab-paclitaxel. Median OS of these patients was 11.2 months. Similarly to PDAC, adjuvant therapy significantly prolonged the median OS of patients with PASC [7, 9, 10]. In our cohort, median OS after resection was 9.2 months (Fig. null). Five out of 8 patients underwent additional procedures such as concomitant vascular resection and adjacent organ resection in an attempt to achieve R0 resection. Contrarily to previous reports, resection status did not positively affect the OS in our cohort, but the small sample size precluded to draw any definitive conclusions. Interestingly, 40 months of DFS was achieved in one patient (case 5) with R1 resection who received adjuvant chemotherapy with gemcitabine. Two recent European series [5, 6] compared PASC with PDAC and reported significantly poorer survival for PASC. The poor prognosis observed in our cohort is consistent with these findings and supports the previously described aggressive nature of PASC.

A recent study reported significantly improved outcomes of PASC patients who received neoadjuvant therapy compared with chemotherapy alone (no information available about the type of chemotherapy) or pancreatic resection alone [8]. In our cohort, neoadjuvant chemotherapy was initiated in 2 patients (case 9, 10). However, surgery was not possible due to disease progression at follow-up reassessment for both patients. More research and data are needed to determine what kind of PASC patients are good candidates for neoadjuvant chemotherapy. Studies on PDAC have showed that neoadjuvant chemotherapy resulted in improved R0 resection rates and OS in patients with borderline resectability [21]. We can assume that PASC patients with arterial contact and/or venous invasion might also be good candidates for neoadjuvant therapy, given its more aggressive behavior. In addition, considering the large number of patients with early postoperative recurrence, it may be possible to select patients with good surgical indications by evaluating the effect of neoadjuvant therapy.

Recurrence occurs usually early after resection, affecting mainly the liver, lung or retroperitoneum, and malignant ascites is also frequent [22–24]. In our series, recurrence occurred early (median DFS: 2.8 months, Fig. 4), in the liver (*n* = 4) and locally in the pancreas (*n* = 1). Malignant ascites as the first symptom of recurrence was not observed in our cohort. Of the 7 patients who had a recurrence, 3 had R1 resections and 5 had lymph node involvement. The median preoperative CA19-9 of the 3 patients with early recurrence (less than 3 months) was 137 U/ml (IQR 16 − 11’000).

Tumor size, lymph node involvement, presence of metastases, high CA19-9 levels, advanced age, location in the distal pancreas, positive surgical margin, the impossibility of surgical resection and the non-administration of adjuvant treatment were described as factors associated with poor survival in PASC series [5, 22, 24]. Due to the small number of patients included in this cohort study, it was not possible to assess prognostic factors of survival.

Some limitations of the study need to be mentioned. As this was a retrospective study, some data were missing, but efforts were made to obtain some missing data by contacting the family doctor or other hospitals involved in the patient management to have as complete files as possible. Moreover, this study included a small number of patients (*n* = 12) due to the rarity of the disease, which precluded multivariable analysis or certain comparisons. However, this series presents detailed clinicopathological characteristics and precise survival data of patients treated for PASC. The granularity of the presented data brings important information on this rare disease.

## Conclusion

Patients with PASC were most often diagnosed with a tumor at an advanced stage (9/12 > stage I). PASC was found in this cohort to have short survival with a high recurrence rate. Although our sample size did not allow us to demonstrate a clear survival benefit of resection, surgical resection remains justified in appropriately selected patients.　Future studies are needed to determine which treatment option is optimal and when neoadjuvant treatment should be administered.

PD: Pancreatoduodenectomy; DP: Distal Pancreatectomy; PV: portal vein; SMV: superior mesenteric vein; LRV: left renal vein. 

## Data Availability

Available on request from the corresponding author.

## References

[1] Fitzgerald TL, Hickner ZJ, Schmitz M, Kort EJ (2008) Changing incidence of pancreatic neoplasms: a 16-year review of statewide tumor registry. Pancreas 37(2):134–138. 10.1097/MPA.0b013e318163a329 18665072 10.1097/MPA.0b013e318163a329

[2] Hsu JT, Yeh CN, Chen YR, Chen HM, Hwang TL, Jan YY, Chen MF (2005) Adenosquamous carcinoma of the pancreas. Digestion 72(2–3):104–108. 10.1159/000088364 16172546 10.1159/000088364

[3] Toshima F, Inoue D, Yoshida K, Yoneda N, Minami T, Kobayashi S, Ikdeda H, Matsui O, Gabata T (2016) Adenosquamous carcinoma of pancreas: CT and MR imaging features in eight patients, with pathologic correlations and comparison with adenocarcinoma of pancreas. Abdom Radiol (NY) 41(3):508–520. 10.1007/s00261-015-0616-4 27039322 10.1007/s00261-015-0616-4

[4] WHO classification of tumours, 5th Edition, digestive system tumours (2019) ;1

[5] Kaiser J, Hinz U, Mayer P, Hank T, Niesen W, Hackert T, Gaida MM, Buchler MW, Strobel O (2021) Clinical presentation and prognosis of adenosquamous carcinoma of the pancreas - Matched-pair analysis with pancreatic ductal adenocarcinoma. Eur J Surg Oncol 47(7):1734–1741. 10.1016/j.ejso.2021.02.011 33622577 10.1016/j.ejso.2021.02.011

[6] Braun R, Klinkhammer-Schalke M, Zeissig SR, van Kleihus K, Bolm L, Honselmann KC, Petrova E, Lapshyn H, Deichmann S, Abdalla TSA, Heckelmann B, Bronsert P, Zemskov S, Hummel R, Keck T, Wellner UF (2022) Clinical outcome and prognostic factors of pancreatic adenosquamous carcinoma compared to ductal adenocarcinoma-results from the german cancer registry group. Cancers (Basel) 14(16). 10.3390/cancers14163946 10.3390/cancers14163946 PMC9406158 36010939

[7] Lv SY, Lin MJ, Yang ZQ, Xu CN, Wu ZM (2022) Survival analysis and prediction model of ASCP based on SEER database. Front Oncol 12:909257. 10.3389/fonc.2022.909257 35814413 10.3389/fonc.2022.909257 PMC9263703

[8] Hue JJ, Katayama E, Sugumar K, Winter JM, Ammori JB, Rothermel LD, Hardacre JM, Ocuin LM (2021) The importance of multimodal therapy in the management of nonmetastatic adenosquamous carcinoma of the pancreas: Analysis of treatment sequence and strategy. Surgery 169(5):1102–1109. 10.1016/j.surg.2020.11.026 33376004 10.1016/j.surg.2020.11.026

[9] Shi Y, Wang X, Wu W, Xie J, Jin J, Peng C, Deng X, Chen H, Shen B (2020) Prognostic analysis and influencing serum biomarkers of patients with resectable pancreatic adenosquamous cancer. Front Oncol 10:611809. 10.3389/fonc.2020.611809 33520722 10.3389/fonc.2020.611809 PMC7844120

[10] Fang Y, Pu N, Zhang L, Wu W, Lou W (2019) Chemoradiotherapy is associated with improved survival for resected pancreatic adenosquamous carcinoma: a retrospective cohort study from the SEER database. Ann Transl Med 7(20):522. 10.21037/atm.2019.10.12 31807504 10.21037/atm.2019.10.12 PMC6861782

[11] Katz MH, Taylor TH, Al-Refaie WB, Hanna MH, Imagawa DK, Anton-Culver H, Zell JA (2011) Adenosquamous versus adenocarcinoma of the pancreas: a population-based outcomes analysis. J Gastrointest Surg 15(1):165–174. 10.1007/s11605-010-1378-5 21082275 10.1007/s11605-010-1378-5 PMC3023036

[12] Boyd CA, Benarroch-Gampel J, Sheffield KM, Cooksley CD, Riall TS (2012) 415 patients with adenosquamous carcinoma of the pancreas: a population-based analysis of prognosis and survival. J Surg Res 174(1):12–19. 10.1016/j.jss.2011.06.015 21816433 10.1016/j.jss.2011.06.015 PMC3210865

[13] Hester CA, Augustine MM, Choti MA, Mansour JC, Minter RM, Polanco PM, Porembka MR, Wang SC, Yopp AC (2018) Comparative outcomes of adenosquamous carcinoma of the pancreas: an analysis of the national cancer database. J Surg Oncol 118(1):21–30. 10.1002/jso.25112 29878370 10.1002/jso.25112

[14] Amin MB, Greene FL, Edge SB, Compton CC, Gershenwald JE, Brookland RK, Meyer L, Gress DM, Byrd DR, Winchester DP (2017) The Eighth edition AJCC cancer staging manual: continuing to build a bridge from a population-based to a more personalized approach to cancer staging. CA Cancer J Clin 67(2):93–99. 10.3322/caac.21388 28094848 10.3322/caac.21388

[15] Campbell FCA, Duthie F, Feakins R (2019) Dataset for the histopathological reporting of carcinoma of the pancreas, ampulla of Vater and common bile duct. The Royal College of Pathologists, London

[16] Moslim MA, Lefton MD, Ross EA, Mackrides N, Reddy SS (2021) Clinical and histological basis of adenosquamous carcinoma of the pancreas: a 30-year experience. J Surg Res 259:350–356. 10.1016/j.jss.2020.09.024 33190924 10.1016/j.jss.2020.09.024 PMC8902409

[17] Hsu JT, Chen HM, Wu RC, Yeh CN, Yeh TS, Hwang TL, Jan YY, Chen MF (2008) Clinicopathologic features and outcomes following surgery for pancreatic adenosquamous carcinoma. World J Surg Oncol 6:95. 10.1186/1477-7819-6-95 18764955 10.1186/1477-7819-6-95 PMC2543014

[18] Ito T, Sugiura T, Okamura Y, Yamamoto Y, Ashida R, Ohgi K, Sasaki K, Uesaka K (2019) Long-term outcomes after an aggressive resection of adenosquamous carcinoma of the pancreas. Surg Today 49(10):809–819. 10.1007/s00595-019-01807-8 30980180 10.1007/s00595-019-01807-8

[19] Auvray Kuentz M, Hautefeuille V, de Mestier L, Coutzac C, Lecomte T, Nardon V, Artru P, Turpin A, Drouillard A, Malka D, Tran-Minh ML, Trouilloud I, Lièvre A, Williet N, Pernot S, Touchefeu Y, Taieb J, Hammel P, Zaanan A (2023) Chemotherapy in advanced pancreatic adenosquamous carcinoma: a retrospective multicenter AGEO study. Int J Cancer 152(9):1894–1902. 10.1002/ijc.34414 36562310 10.1002/ijc.34414

[20] Yoshida Y, Kobayashi S, Ueno M, Morizane C, Tsuji K, Maruki Y, Mori K, Watanabe K, Ohba A, Furuta M, Todaka A, Tsujimoto A, Ozaka M, Okano N, Yane K, Umemoto K, Kawamoto Y, Terashima T, Tsumura H, Doi K, Shioji K, Asagi A, Kojima Y, Suzuki E, Toshiyama R, Furukawa M, Naganuma A, Suzuki R, Miwa H, Ikeda M, Furuse J (2022) Efficacy of chemotherapy for patients with metastatic or recurrent pancreatic adenosquamous carcinoma: a multicenter retrospective analysis. Pancreatology 22(8):1159–1166. 10.1016/j.pan.2022.09.236 36150984 10.1016/j.pan.2022.09.236

[21] Hajibandeh S, Hajibandeh S, Intrator C, Hassan K, Sehmbhi M, Shah J, Mazumdar E, Kausar A, Satyadas T (2023) Neoadjuvant chemoradiotherapy versus immediate surgery for resectable and borderline resectable pancreatic cancer: meta-analysis and trial sequential analysis of randomized controlled trials. Ann Hepatobiliary Pancreat Surg 27(1):28–39. 10.14701/ahbps.22-052 36536501 10.14701/ahbps.22-052 PMC9947376

[22] Boecker W, Tiemann K, Boecker J, Toma M, Muders MH, Loning T, Buchwalow I, Oldhafer KJ, Neumann U, Feyerabend B, Fehr A, Stenman G (2020) Cellular organization and histogenesis of adenosquamous carcinoma of the pancreas: evidence supporting the squamous metaplasia concept. Histochem Cell Biol 154(1):97–105. 10.1007/s00418-020-01864-y 32170368 10.1007/s00418-020-01864-y PMC7343762

[23] Komatsu H, Egawa S, Motoi F, Morikawa T, Sakata N, Naitoh T, Katayose Y, Ishida K, Unno M (2015) Clinicopathological features and surgical outcomes of adenosquamous carcinoma of the pancreas: a retrospective analysis of patients with resectable stage tumors. Surg Today 45(3):297–304. 10.1007/s00595-014-0934-0 24973941 10.1007/s00595-014-0934-0

[24] Borazanci E, Millis SZ, Korn R, Han H, Whatcott CJ, Gatalica Z, Barrett MT, Cridebring D, Von Hoff DD (2015) Adenosquamous carcinoma of the pancreas: molecular characterization of 23 patients along with a literature review. World J Gastrointest Oncol 7(9):132–140. 10.4251/wjgo.v7.i9.132 26380056 10.4251/wjgo.v7.i9.132 PMC4569590

